# Comparison of Pre-Diagnosis Physical Activity and Its Correlates between Lung and Other Cancer Patients: Accelerometer Data from the UK Biobank Prospective Cohort

**DOI:** 10.3390/ijerph20021001

**Published:** 2023-01-05

**Authors:** Weijiao Zhou, Philip T. Veliz, Ellen M. Lavoie Smith, Weiyun Chen, Rishindra M. Reddy, Janet L. Larson

**Affiliations:** 1 School of Nursing, University of Michigan, Ann Arbor, MI 48109, USA; 2 School of Nursing, Peking University, Beijing 100191, China; 3 School of Nursing, University of Alabama at Birmingham, Birmingham, AL 35294, USA; 4 School of Kinesiology, University of Michigan, Ann Arbor, MI 48109, USA; 5 Department of Thoracic Surgery, University of Michigan Medical Center, Ann Arbor, MI 48109, USA

**Keywords:** pre-diagnosis physical activity, moderate to vigorous physical activity, cancer, lung cancer, accelerometry, survival analysis, UK biobank

## Abstract

Purpose: Physical activity (PA) plays an important role in health outcomes for people with cancer, and pre-diagnosis PA influences PA behaviors after cancer treatment. Less is known about the PA of lung cancer patients, and the strong history of smoking could influence pre-diagnosis levels of PA and place them at risk for health problems. This study aimed to compare pre-diagnosis PA and its correlates in patients with lung cancer and other types of cancer (female breast, colorectal, and prostate cancer) and examine the relationship between pre-diagnosis PA and all-cause mortality. Methods: This study used data from the UK Biobank, which is a national cohort study with accelerometry data. We included 2662 participants and used adjusted linear regressions and survival analyses. Results: Male and female lung cancer groups spent a mean of 78 and 91 min/day in pre-diagnosis moderate to vigorous PA (MVPA), respectively; this is lower than the 3 other types of cancer (*p* < 0.001). Younger age and faster walking pace had a strong association with PA in all the four types of cancer (*p* < 0.01). Smoking status had a strong association with PA in the lung cancer group, while obesity had a strong association with PA in female breast, colorectal, and prostate cancer (*p* < 0.01). Higher levels of pre-diagnosis MVPA (≥1.5 h/day) were associated with a significantly lower all-cause mortality risk. Conclusions: The present study suggests that lung cancer patients are the most inactive population before diagnosis. The identified difference in correlates of PA suggest that cancer-specific approaches are needed in PA research and practices. This study also highlights the importance of high PA for individuals with high cancer risk.

## 1. Introduction

Physical activity (PA) provides numerous health benefits for cancer patients. Physical activity improves health outcomes including cardiovascular fitness, muscle strength, cancer-related fatigue, health-related quality of life, and depression [1,2,3]. Physical activity guidelines for cancer survivors recommend at least 150 min of moderate PA per week [4]. However, only 8–58% of cancer survivors meet the PA recommendations [5,6,7,8]. In this research, we focused on lung cancer and PA because less is known about the PA of lung cancer patients (compared to breast, colorectal, and prostate cancer), and the strong history of smoking could influence other lifestyle behaviors (e.g., PA) and place them at risk for health problems.

Much of the prior research focuses on PA after cancer diagnosis, but it is important to examine the entire continuum. The PA and Cancer Control (PACC) framework proposes six cancer-related time periods: two pre-diagnosis (pre-screening and screening) and four post-diagnosis (pre-treatment, treatment, survivorship, and end of life) [9]. Most existing research focuses on PA during the post-diagnosis period (e.g., before surgery/chemotherapy, during chemotherapy, and after surgery/chemotherapy); less is known about pre-diagnosis PA. It is well established that a higher pre-diagnosis PA is associated with reduced overall/cancer-specific mortality among breast, colorectal, prostate, and lung cancer patients [10,11,12,13,14], and higher pre-diagnosis PA is a strong predictor of post-diagnosis PA [15,16,17]. However, less is known about the actual volume of pre-diagnosis PA and its correlates. Cancer diagnosis is a “Teachable Moment” [18,19]; understanding pre-diagnosis PA and its correlates will be helpful in identifying patients with high risk of physical inactivity. Information about the volume of PA prior to diagnosis and its relationship to health outcomes can be used in patient education materials and in PA interventions to motivate behavior change.

Lung cancer is the second most common cancer and the leading cause of cancer-related death worldwide [20]. However, individuals with lung cancer are understudied compared with breast, colorectal, and prostate cancer [2]. A systematic review synthesized evidence regarding the factors influencing PA in lung cancer survivors, and the findings were derived mainly from qualitative studies or quantitative studies with self-reported PA measures [21]. Self-reported data are subject to response bias (e.g., imprecise recall, influence of social desirability) [22] and is less accurate compared to data obtained using objective measures, such as accelerometers [23]. Research is needed to examine pre-diagnosis PA behaviors using objective measures in lung cancer patients to characterize their PA features compared to patients with other cancer types (e.g., breast, colorectal, and prostate cancer). This information will be helpful in determining if the same approach to promote PA is appropriate for all cancer types or if a cancer-specific approach is needed.

Several socio-demographic and health-related characteristics have been identified as correlates of PA in cancer survivors (e.g., younger age, lower BMI, fewer comorbidities) [24,25,26,27], but few studies compared patients with different types of cancer using the same sampling frame and measures. To our knowledge, only a few studies compared PA correlates, but they focused on post-diagnosis PA among breast, colorectal, and prostate cancer patients and used self-reported PA measures [6,15]. Less is known about PA features of lung cancer patients compared to other cancer patients. To fill in this research gap, we conducted a secondary analysis of the accelerometer data from a national cohort study (UK Biobank). The aims were to: (1) compare pre-diagnosis PA of patients with lung cancer to patients with other types of cancer (female breast, colorectal, and prostate cancer); (2) compare correlates of pre-diagnosis PA between patients with lung cancer and other types of cancer; and (3) examine the relationship between pre-diagnosis PA and all-cause mortality after lung, female breast, colorectal, and prostate cancer diagnosis.

## 2. Methods

### 2.1. Data Source and Participants

This study used data collected from the UK Biobank, which is a national cohort study in the UK. Demographic and health-related data were collected from 500,000 participants (aged 40–69 years) between 2006 and 2010, with a reassessment of 20,000 participants between 2012 and 2013 (the most recent data were used—this is referred to as baseline data for the purpose of this current study). Between 2013 and 2015, participants were re-contacted and invited to wear an accelerometer (Axivity AX3 wrist-worn triaxial accelerometer, a commercial version of the Open Movement AX3 designed by Open Lab, Newcastle University, UK) if they provided a valid email address at the baseline assessment. Of the total recruited 500,000 UK Biobank sample, 236,519 participants were asked to join the accelerometer study to obtain objectively measured PA data under free-living conditions. A total of 106,053 agreed to wear a PA monitor (response rate to invitations = 44.8%), and 103,720 participants returned data between 2013 and 2015 [28]. Data from cancer and death registries were linked to the UK Biobank cohort to provide information on cancer diagnoses and death. The UK Biobank protocol was approved by the North-West Multicenter Research Ethics Committee.

**Figure 1:**
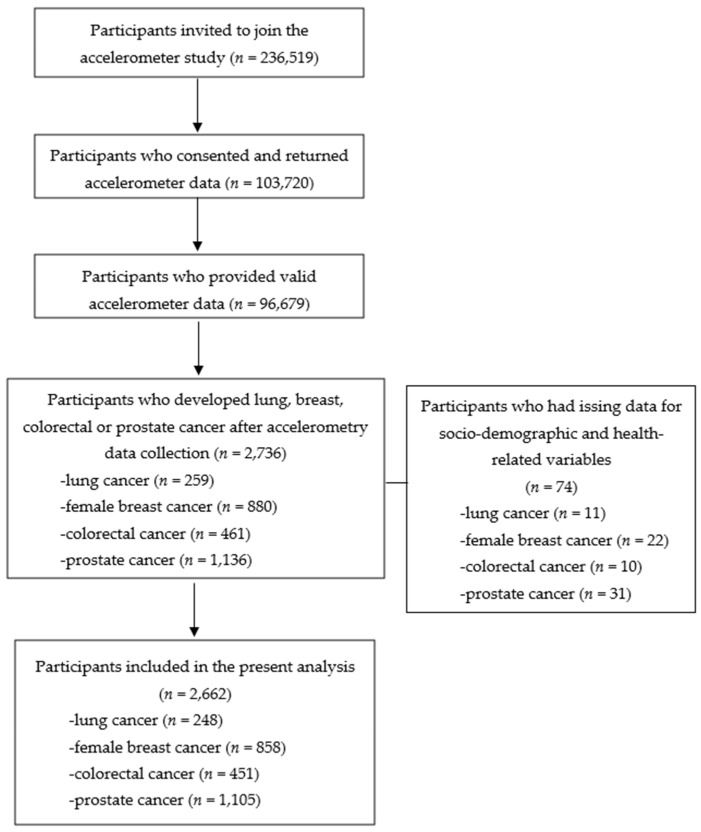
Flowchart of study sample.

We included participants if they: (1) were diagnosed with primary lung cancer, female breast cancer, colorectal cancer, or prostate cancer after completing accelerometer data collection (see Supplementary Table S1 for ICD 9 and ICD 10 codes); (2) had valid accelerometer data (≥3 days of wear time) [28]; and (3) had no missing values for socio-demographic and health-related variables (see below). A total of 2662 participants were included (lung cancer = 248, female breast cancer = 858, colorectal cancer = 451, prostate cancer = 1105) (see Figure 1 for details).

### 2.2. Measures

Physical activity was collected with the Axivity AX3 wrist-worn triaxial accelerometer between 2013 and 2015. The participants were instructed to do the following: (1) start wearing the accelerometer device immediately after receiving it, (2) wear it for seven continuous days on their dominant wrist, (3) carry on with their normal activities, and (4) mail the device back to the research center, in a pre-paid envelope, after the seven-day monitoring period [28]. The raw accelerometer data were calibrated, and wear-time periods were identified using the UK Biobank preprocessing methods described by Doherty et al. [28]. Accelerometer-based summary measures in the dataset included the total mean acceleration/24 h (vector magnitude in milligravity units = mg) and time spent in sedentary, light, and moderate-to-vigorous PA (MVPA). The proportion of time spent in moderate and vigorous PA was defined as the proportion of time spent in accelerations of 101–425 and >425 milligravity, respectively [29]. The same cut-points have been used by others to calculate time spent in different intensities of PA for the UK Biobank accelerometry data [28,30,31,32].

Health-related factors were collected at baseline (2006–2010 and 2012–2013). (1) Self-reported overall health was rated as excellent, good, fair, or poor. (2) Self-reported comorbidities were measured using a 13-item comorbidity check list. For the purposes of this research, we analyzed data from patients with the most common cardiovascular and pulmonary comorbidities (heart attack, angina, stroke, hypertension, COPD, and asthma) and diabetes. The number of comorbidities ranged from 0 to 7. (3) Self-reported walking pace was measured using an item “How would you describe your usual walking pace?” with response options of slow, steady/average, or brisk. Participants could access further information which defined a slow pace as less than three miles per hour, a steady/average pace as between three and four miles per hour, and a brisk pace as more than four miles per hour. (4) Grip strength was assessed in each hand using a hydraulic hand dynamometer (Jamar J00105, Lafayette, IN, USA), which can measure isometric grip force up to 90 kg [33]. Grip strength was measured in both hands and the highest value was used for analyses. (5) Self-reported anxiety and depression was measured using a short version of the Patient Health Questionnaire (PHQ-9) and Generalized Anxiety Disorder [34,35,36]. Participants were asked “How often have you felt down, depressed, or hopeless”, “How often have you had little interest or pleasure in doing things”, “How often have you felt tense, fidgety, or restless”, and “How often have you felt tired or had little energy” over the past two weeks, with response options of “not at all = 1”, “several days = 2”, “more than half the days = 3”, and “nearly every day = 4”. Scores ranged from 4 to 16, in which higher scores indicated more severe symptoms.

Socio-demographic characteristics were collected at baseline (2006–2010 and 2012–2013), including age, sex, ethnicity (white/non-white), Townsend Index of deprivation (high scores indicated higher levels of socioeconomic deprivation) [37], body mass index (BMI, underweight/normal/overweight/obese), smoking status (never/previous/current smoker), and alcohol drinking frequency (≤1–3 times/month, 1–4 times/week, daily, or almost daily).

Date of cancer diagnosis and death were linked to the UK Biobank dataset. The included participants were diagnosed with lung/female breast/colorectal/prostate cancer between 2013 and 2020 (at 4 days–6.5 years after accelerometer data collection). We followed participants from their date of cancer diagnosis to their date of death as provided by UK Biobank’s linkage to death registration data or to the latest follow-up date for mortality data (21 March 2021) if they did not have a death record.

### 2.3. Data Analysis

Stata SE 17.0 software (StataCorp LLC., College Station, TX, USA) was used for data analysis. Descriptive statistics (percentages for categorical variables and mean and standard deviation for continuous variables) were calculated for socio-demographic, health-related characteristics, and accelerometer-measured PA in each type of cancer, stratified by sex. We compared the socio-demographic, health-related characteristics, and accelerometer-measured PA among different cancer groups stratified by sex. Chi-square tests of independence were used for categorical variables, and ANOVA tests were used for continuous variables. A *p*-value of less than 0.01 was considered statistically significant for all analyses.

To address the three study aims (see Introduction Section), we used linear regressions and survival analyses. Aim 1: Linear regression was used to compare time spent in MVPA between patients with lung cancer and other types of cancer, stratified by sex (independent variable = type of cancer; dependent variable = MVPA). The linear regression models included both unadjusted and adjusted estimates that control for socio-demographic characteristics. The unadjusted and adjusted coefficients and 95% confidence intervals (95% CI) were reported.

Aim 2: Linear regressions were used to examine the correlates of PA for each type of cancer (independent variable = sex, age, race, Townsend Index of Deprivation, BMI, smoking status, alcohol drinking frequency, overall health rating, number of comorbidities, walking pace, grip strength, and anxiety and depression; dependent variable = MVPA). The linear regression models included both unadjusted and adjusted estimates that control for other socio-demographic and health-related characteristics. The unadjusted coefficients, adjusted coefficients, 95% CI, and adjusted standardized coefficients were reported.

Aim 3: Survival analyses (Cox regressions) were used to assess the potential impact of time spent in MVPA on all-cause mortality. We used Cox regressions to model time-to-death as a function of time spent in MVPA per day and controlled for socio-demographics, cancer types, and comorbidities. The unadjusted and adjusted Hazard Ratio (HR) and 95% CI for all-cause mortality were reported. Interaction analysis was performed to explore whether cancer types modified the association between MPVA and all-cause mortality.

## 3. Results

### 3.1. Characteristics of Participants

A total of 2662 participants were included in this study; they developed lung, female breast, colorectal, or prostate cancer after accelerometry data collection and met other criteria for inclusion. Participants in the lung cancer group were more likely to be current smokers and report brisk walking pace, compared to people with other types of cancer (*p* < 0.01). In addition, participants in the female lung cancer group were older than the female breast cancer and female colorectal cancer groups (*p* < 0.001). Participants in the male lung cancer group reported less frequent alcohol consumption, worse overall health, and more comorbidities and had lower grip strength compared to the prostate cancer and male colorectal cancer groups (*p* < 0.01) (see Table 1 for detail).

When stratifying by sex, the total acceleration was 25.31 mg/day and 22.87 mg/day in female and male lung cancer groups, respectively; this was lower than other types of cancer (*p* < 0.01). The time spent in MVPA per day was 91 and 78 min/day in female and male lung cancer groups, respectively; this was lower than other types of cancer (*p* < 0.001) (see Table 1).

### 3.2. Comparison of Pre-Diagnosis PA

In the unadjusted linear regression models of females, participants in the breast and colorectal cancer group spent 15.68 (95% CI: 7.58, 23.78, *p* < 0.001) and 10.93 (95% CI: 1.18, 20.67, *p* < 0.05) more minutes in MVPA, respectively, compared with participants in the lung cancer group. The difference disappeared with adjustment for socio-demographic characteristics (see Table 2 for details).

In the unadjusted linear regression models for males, participants in the colorectal and prostate cancer group spent 13.46 (95% CI: 3.72, 23.19, *p* < 0.01) and 23.54 (95% CI: 15.07, 32.01, *p* < 0.001) more minutes in MVPA, respectively, compared with participants in the lung cancer group. With adjustment for socio-demographic characteristics, the difference between colorectal and lung cancer was not statistically significant, but participants in the prostate cancer group still showed 15.32 more minutes in MVPA per day compared with lung cancer (95% CI: 7.11, 23.53, *p* < 0.001) (see Table 2 for details).

### 3.3. Comparison of Correlates of Pre-Diagnosis PA

In the unadjusted linear regression models, higher pre-diagnosis PA was associated with younger age, lower BMI, better self-rated health, fewer comorbidities, and faster self-rated walking pace in all the four types of cancer (*p* < 0.01). In the lung cancer group, higher pre-diagnosis PA was also associated with never smoking, more frequent alcohol consumption, and lower anxiety and depression (*p* < 0.01) (see Table 3).

In the adjusted linear regression models, we compared the relative strength of the correlates on PA in each type of cancer. Younger age and faster walking pace had a strong association with PA in all the four types of cancer (*p* < 0.01). In addition, smoking status had a strong negative association with PA in the lung cancer group, while obesity had a strong negative association with PA in female breast, colorectal, and prostate cancer (*p* < 0.01) (see Table 3 and Supplementary Table S2).

### 3.4. Survival Analysis

A total of 2662 cancer cases diagnosed between 2013 and 2020 were followed up to 2021 (up to 7.4 years). There were 426 deaths among 2661 participants during the follow-up period (1 participant died on the same day of cancer diagnosis). Cox regression analyses showed that higher levels of pre-diagnosis MVPA (≥1.5 h/day) were associated with a significantly lower all-cause mortality risk after cancer diagnosis (HR = 0.57–0.68, *p* < 0.01) (see Table 4). However, no significant difference for all-cause mortality was found between “MVPA = 1–1.5 h/day” and “MVPA less than 1 h/day” (*p* > 0.05). Compared to lung cancer, patients with other types of cancer had a significantly lower all-cause mortality risk (HR = 0.08–0.31, *p* < 0.001) (see Table 4). There was no significant interaction between cancer types and MVPA on all-cause mortality (see Supplementary Table S3). The association between PA and mortality was not significantly different between different cancer types.

## 4. Discussion

To our knowledge, this study is the first to compare pre-diagnosis PA and its correlates between patients with lung cancer and other types of cancer (e.g., breast, colorectal, and prostate cancer) using a national cohort dataset with objective measures. The present study found that lung cancer patients were the most physically inactive population before diagnosis compared to female breast and colorectal cancer, but this difference could be explained by socio-demographic characteristics. This study identified correlates of pre-diagnosis PA among patients with each type of cancer and detected differences between lung cancer and other types of cancer (female breast, colorectal, and prostate cancer). Specifically, smoking status was negatively associated with pre-diagnosis PA in lung cancer only, while obesity was negatively associated with pre-diagnosis PA in the female breast, colorectal, and prostate cancer groups. Furthermore, the present study found that higher MVPA before diagnosis (≥1.5 h/day) was associated with a lower all-cause mortality risk after lung, female breast, colorectal, and prostate cancer diagnosis.

### 4.1. Pre-Diagnosis MVPA

The present study found that lung cancer patients had lower pre-diagnosis MVPA, and their lower pre-diagnosis MVPA could be explained by socio-demographics. This was also observed by Sweegers et al. (2019) for post-diagnosis MVPA [38]. Sweegers et al. (2019) compared lung cancer survivors’ PA with breast cancer survivors’ data on PA and found no significant difference after controlling for age, sex, and smoking status.

The pre-diagnosis MVPA in the present study appears high in comparison to previous estimates [8,38] and the recommended MVPA guidelines (150 min/week) [4]. This can be explained by differences in the data reduction methods for PA. The moderate and vigorous intensity PA data were generated by applying thresholds to the raw acceleration data acquired from a wrist-worn device [29]. These results are not comparable to data acquired by devices mounted at the hip and data reported as activity counts, but the results are comparable to other studies that used similar methods even with different devices. Other studies using the UK Biobank data and the same cut-points found that cancer survivors and patients with chronic diseases spent similar amount of time in MVPA compared to this current study [31,32]. In addition, these results cannot be used to determine if subjects met PA guidelines, because the guidelines were established using self-reported measures of PA behavior that are not comparable to objectively measured PA.

### 4.2. Correlates of PA

The present study found two strong correlates of pre-diagnosis MVPA in all four types of cancer: age and walking speed. Age is a well-established predictor of PA. Prior studies with accelerometers reported a negative association between age and PA in general cancer survivors [8,38] and patients with breast [17,27], colorectal [26], and lung cancer [39]. Walking speed/pace is considered to be a “vital sign” and indicative of functional status and health outcomes [40,41], and it is a recognized predictor of daily PA in older adults [42]. This study extends the finding from prior studies of post-diagnosis PA or PA in older adults and identified walking speed as a predictor of pre-diagnosis MVPA.

The differences in PA correlates between cancer types were observed by prior studies with self-reported PA measures [6,15]. The present study identified cancer-specific PA correlates: smoking status and obesity. Smoking was a strong correlate of pre-diagnosis MVPA in lung cancer patients only. This finding is consistent with previous studies in post-diagnosis PA of lung cancer patients [39]. However, a few studies of a mixed cancer population reported inconsistent findings regarding whether smoking status was associated with PA [5,38]. The inconsistent findings could be potentially explained by the proportion of lung cancer patients in the mixed cancer population, given that the association between smoking status and PA was predominantly present in lung cancer [38]. Obesity was a strong correlate of pre-diagnosis MVPA in the female breast, colorectal, and prostate cancer patients. Similar results were also found in the PA of general cancer survivors [5,38], colorectal cancer survivors [26], and breast cancer survivors [27]. However, obesity was not a strong correlate of MVPA in lung cancer patients [39].

The present study supports the finding of prior studies that focused on only one of these cancer types, and it also helps to explain the inconsistent findings of prior studies with mixed cancer populations. Future studies may consider the similarity within and differences between cancer types when merging a mixed cancer population. The identified differences in PA and its correlates between cancer types suggest that cancer-specific approaches are needed to identify patients at high risk of physical inactivity. For example, PA interventions in lung cancer could target smokers, who are at higher risk for low PA than non-smokers. Given the correlation between smoking and PA, behavioral change interventions might be more effective if smoking cessation and PA promotion strategies were combined for smokers.

### 4.3. Pre-Diagnosis PA and All-Cause Mortality

The present study, with a national cohort and accelerometer data, confirms the findings from previous studies of self-reported PA measures [2] and supports the benefits of pre-diagnosis MVPA on improved survival outcomes. Recent meta-analyses found that higher pre-diagnosis PA was protective against cancer-specific mortality following breast, colorectal, and lung cancer and against all-cause mortality among breast, colorectal, and prostate cancer [2], but the majority of studies used self-reported PA measures, and few studies examined the dose–response effects of MVPA on mortality. The present study found that engaging in less than 1.5 h/day of MPVA may not provide significant benefits for survival, but this finding should be interpreted with caution considering the differences in PA measures derived from wrist-worn vs. hip-worn devices [43]. In addition, the present study also suggests that the effect of MVPA on survival is not modified by different cancer types. Patients with each type of cancer may receive the same survival benefits from the same PA levels. In addition to mortality, a previous study also found that pre-diagnosis PA is a strong influencing factor of early recurrences of slow-growing cancer [44], and it is also an important predictor of post-diagnosis PA [15]. The above evidence highlights the need for PA promotion among people with higher cancer risks.

### 4.4. Limitations

This study has major strengths that include a large sample from a national cohort and objective measures of PA, but it also has several limitations. First, the accelerometer data were not collected immediately prior to the cancer diagnosis: there is a time interval (4 days–6.5 years) between PA assessment and cancer diagnosis. Thus, this may not be representative of participants’ overall pre-diagnosis PA levels. We assumed that PA remained roughly the same before cancer diagnosis while controlling for age. Second, we did not have information on cancer stage and cancer treatments, which are confounding variables in PA and survival. Third, accelerometer study participants represent a subset of the UK Biobank participants who were willing and able to join a study on the objective measurement of PA but may not be representative of the broader UK population [31]. Fourth, time spent in MVPA is high in this study, but we could not determine if people meet PA guidelines based on the MVPA data considering the nature of data produced by wrist-worn accelerometers.

## 5. Conclusions

Understanding pre-diagnosis PA and its correlates is helpful in promoting PA and ultimately improving health outcomes in cancer patients. This study is the first to compare objectively measured pre-diagnosis MVPA and its correlates between patients with lung cancer and other common cancers, and the first to examine the dose–response relationship between MVPA and all-cause mortality, using a national cohort. Lung cancer patients are the most physically inactive population before diagnosis compared to female breast and colorectal cancer, but this difference could be explained by socio-demographic characteristics. Age and walking speed were strong PA correlates in all four types of cancer, while smoking status was a unique correlate in lung cancer. This study confirms the finding of prior studies that focused on only one of these cancer types and suggests cancer-specific approaches in PA research and practice, especially for lung cancer. Higher pre-diagnosis MVPA (>1.5 h) is associated with a lower all-cause mortality risk, which highlights the importance of high PA for individuals with high cancer risk.

## Figures and Tables

**Table 1:** Description of study population by tumor type (stratified by sex) (*n* = 2662).

Characteristics	Female				Male			
	Lung (*n* = 129)	Breast (*n* = 858)	Colorectal (*n* = 194)	*p* Value ^a^	Lung (*n* = 119)	Prostate (*n* = 1105)	Colorectal (*n* = 257)	*p* Value ^b^
Socio-demographics								
Age at accelerometer study, mean (SD)	66.09 (5.81)	62.82 (7.50)	65.74 (6.59)	<0.001	68.44 (5.89)	67.95 (5.66)	66.94 (6.50)	<0.05
Age at cancer diagnosis, mean (SD)	68.81 (5.96)	65.33 (7.64)	68.25 (6.77)	<0.001	71.32 (5.97)	69.72 (5.64)	69.52 (6.82)	<0.05
White race, %	97.67	97.44	96.91	0.894	97.48	97.92	98.83	0.571
Townsend Index of deprivation, mean (SD)	−0.96 (3.12)	−1.71 (2.73)	−1.41 (3.02)	<0.05	−1.31 (3.13)	−2.05 (2.60)	−1.78 (2.78)	<0.01
BMI, % Underweight	0.78	0.93	1.03	0.226	0.00	0.09	0.00	<0.05
Normal	35.66	40.79	39.18		23.53	27.15	24.51	
Overweight	35.66	40.09	36.60		42.86	53.57	52.53	
Obese	27.91	18.18	23.20		33.61	19.19	22.96	
Smoking status, %				<0.001				<0.001
Never	27.13	57.46	45.88		15.97	48.05	45.91	
Previous	60.39	36.25	51.03		58.82	46.06	44.36	
Current	22.48	6.29	3.09		25.21	5.88	9.73	
Alcohol drinking frequency, %				0.143				<0.01
≤1–3 times/month	37.21	29.37	36.08		27.73	16.20	14.01	
1–4 times/week	41.09	49.18	41.75		41.18	52.94	48.64	
Daily or almost daily	21.71	21.45	22.16		31.09	30.86	37.35	
Health-related characteristics								
Overall health rating, %				<0.05				<0.001
Excellent	10.85	19.93	20.10		10.08	21.54	16.73	
Good	63.57	64.69	64.43		50.42	60.54	61.09	
Fair	18.60	12.94	13.40		32.77	16.65	19.46	
Poor	6.98	2.45	2.06		6.72	1.27	2.72	
No. of comorbidities, %				0.149				<0.001
0	56.59	64.57	59.28		40.34	57.74	54.09	
1	31.78	28.90	31.96		35.29	33.57	32.30	
2+	11.63	6.53	8.76		24.37	8.69	13.62	
Self-reported walking pace, %				<0.001				<0.001
Slow	15.50	478.	8.76		11.76	3.35	6.61	
Steady or average	54.26	51.86	47.94		53.78	48.69	52.14	
Brisk	30.23	43.36	43.30		34.45	47.71	41.25	
Grip strength in kg, mean (SD)	24.32 (6.64)	25.08 (6.29)	24.62 (5.81)	0.339	38.93 (8.32)	41.09 (8.33)	39.87 (8.08)	<0.01
Anxiety and depression, mean (SD)	5.48 (2.12)	5.26 (1.94)	5.24 (1.72)	0.456	5.03 (1.58)	5.02 (1.55)	4.99 (1.64)	0.930
Accelerometer measured PA								
Total acceleration, mean (SD)	25.31 (7.79)	27.50 (7.32)	26.57 (7.11)	<0.01	22.87 (6.83)	26.91 (8.74)	24.82 (7.47)	<0.001
MVPA (min/day), mean (SD)	91.05 (44.16)	106.74 (43.37)	101.98(45.02)	<0.001	77.95 (36.04)	101.49 (45.72)	91.41 (44.11)	<0.001
Moderate PA (min/day), mean (SD)	88.72 (41.90)	103.53 (40.99)	99.65 (43.50)	<0.001	75.78 (34.49)	97.40 (43.06)	88.27 (42.11)	<0.001
Vigorous PA (min/day), mean (SD)	2.33 (4.37)	3.20 (4.86)	2.33 (3.20)	<0.05	2.18 (4.04)	4.09 (5.89)	3.14 (4.28)	<0.001

Note: ^a^ Comparison between female lung cancer, female breast cancer, and female colorectal cancer. ^b^ Comparison between male lung cancer, prostate cancer, and male colorectal cancer. SD: standard deviation; BMI: body mass index; MVPA: moderate to vigorous physical activity.

**Table 2:** Comparison of time spent in moderate to vigorous physical activity between different type of cancer (min/day) (stratified by sex).

	Female (*n* = 1181)		Male (*n* = 1481)	
	Unadjusted Coefficient b (95% CI)	Adjusted Coefficient ^a^ b (95% CI)	Unadjusted Coefficient b (95% CI)	Adjusted Coefficient ^a^ b (95% CI)
Type of cancer				
Lung cancer	0.00 (reference)	0.00 (reference)	0.00 (reference)	0.00 (reference)
Female breast cancer	15.68 (7.58, 23.78) ***	5.62 (–2.26, 13.50)	−	−
Colorectal cancer	10.93 (1.18, 20.67) *	6.37 (−2.90, 15.64)	13.46 (3.72, 23.19) **	6.33 (−2.97, 15.64)
Prostate cancer	−	−	23.54 (15.07, 32.01) ***	15.32 (7.11, 23.53) ***
Age at accelerometer study		−1.61 (−1.94, −1.27) ***		−2.41 (−2.78, −2.04)
White race (reference: non-white)		−0.49 (−15.38, 14.39)		−7.72 (−23.36, 7.91)
Townsend Index of deprivation		−0.31 (−1.15, 0.53)		−0.32 (−1.14, 0.49)
BMI				
Underweight		8.65 (−15.84, 33.15)		64.43 (−17.77, 146.613)
Normal		0.00 (reference)		0.00 (reference)
Overweight		−9.44 (−14.75, −4.14) ***		−11.35 (−16.49, −6.20) ***
Obese		−21.74 (−28.27, −15.21) ***		−24.20 (−30.54, −17.85) ***
Smoking status				
Never		0.00 (reference)		0.00 (reference)
Previous		1.08 (−3.97, 6.13)		5.68 (1.04, 10.32) *
Current		−14.26 (−23.61, −4.90) **		−11.73 (−20.18, −3.28) **
Alcohol drinking frequency				
≤1–3 times/month		0.00 (reference)		0.00 (reference)
1–4 times/week		9.43 (3.94, 14.92) **		6.60 (0.55, 12.65) *
Daily or almost daily		12.66 (5.94, 19.38) ***		7.63 (1.11, 14.16) *

Note: ^a^ adjusted for age, race, Townsend Index of deprivation, BMI, smoking status, and alcohol drinking frequency. * *p* < 0.05, ** *p* < 0.01, *** *p* < 0.001. CI: confidence interval; BMI: body mass index.

**Table 3:** Comparison of correlates of time spent in MVPA (min/day) between lung, female breast, colorectal, and prostate cancer.

	**Lung Cancer (*n* = 248)**		**Breast Cancer (*n* = 858; Only Females)**	
	**Unadjusted Coefficient** **b (95% CI)**	**Adjusted Coefficient** **b (95% CI)**	**Unadjusted Coefficient** **b (95% CI)**	**Adjusted Coefficient** **b (95% CI)**
Male (reference: female)	−13.10 (−23.23, −2.97) *	1.36 (−12.66,15.39)	−	−
Age at accelerometer study	−2.29 (−3.10, −1.48) ***	−2.35 (−3.22, −1.48) ***	−1.57 (−1.94, −1.19) ***	−1.50 (−1.90, −1.11) ***
White (reference: non−white)	−8.80 (−42.16, 24.55)	−3.04 (−32.88,26.80)	−5.86 (−24.25, 12.53)	−3.31 (−20.64, 14.01)
Townsend Index of Deprivation	−2.14 (−3.76, −0.51) *	−1.23 (−2.80, 0.36)	0.28 (−0.79, 1.34)	0.65 (−0.36, 1.65)
BMI				
Underweight	−54.23 (−134.20, 25.73)	−47.59 (−120.05, 24.87)	27.86 (−1.76, 57.48)	19.05 (−9.05, 47.15)
Normal	0.00 (reference)	0.00 (reference)	0.00 (reference)	0.00 (reference)
Overweight	−8.96 (−21.21, 3.30)	−3.86 (−15.02, 7.30)	−14.31 (−20.60, −8.02) ***	−8.48 (−14.60, −2.35) **
Obese	−19.79 (−32.76, −6.82) **	−8.36 (−21.19, 4.47)	−25.54 (−33.51, −17.56) ***	−14.99 (−22.94, −7.04) ***
Smoking status				
Never	0.00 (reference)	0.00 (reference)	0.00 (reference)	0.00 (reference)
Previous	−18.09 (−30.57, −5.62) **	−7.06 (−18.83, 4.70)	1.09 (−5.07, 7.26)	3.71 (−2.08, 9.50)
Current	−34.96 (−49.55, −20.37) ***	−26.43 (−40.30, −12.56) ***	−8.62 (−20.82, 3.58)	−8.88 (−20.24, 2.47)
Alcohol drinking frequency				
≤1–3 times/month	0.00 (reference)	0.00 (reference)	0.00 (reference)	0.00 (reference)
1–4 times/week	16.51 (4.70, 28.33) **	1.90 (−9.12, 12.92)	11.43 (4.70, 18.17) **	7.71 (1.37, 14.06) *
Daily or almost daily	17.45 (4.23, 30.67) *	8.97 (−3.16, 21.11)	11.78 (3.58, 19.99) **	9.55 (1.71, 17.39) *
Overall health rating				
Excellent	0.00 (reference)	0.00 (reference)	0.00 (reference)	0.00 (reference)
Good	−3.87 (−20.54, 12.81)	0.93 (−14.05, 15.91)	−5.21 (−12.58, 2.15)	−0.55 (−7.71, 6.61)
Fair	−21.98 (−40.20, −3.75) *	−1.36 (−19.16, 16.43)	−18.80 (−29.06, 8.54) ***	−6.91 (−17.48, 3.65)
Poor	−36.90 (−61.28, −12.52) **	−15.35 (−43.42, 12.71)	−33.90 (−53.36, −14.43) **	−20.77 (−41.01, −0.53) *
No. of comorbidities	−12.87 (−18.11, −7.64) ***	−3.10 (−9.02, 2.81)	−8.36 (−12.84, −3.88) ***	−2.40 (−6.80, 2.02)
Self-reported walking pace				
Slow	0.00 (reference)	0.00 (reference)	0.00 (reference)	0.00 (reference)
Steady or average	25.64 (1−.99, 40.29) **	11.36 (−3.54, 26.26)	26.85 (13.41, 40.28) ***	16.36 (2.53, 30.18) *
Brisk	43.24 (27.62, 58.86) ***	23.06 (6.52, 39.60) ***	44.12 (30.57, 57.67) ***	29.08 (14.84, 43.33) ***
Grip strength (kg)	−0.08 (−0.57, 0.41)	−0.60 (−1.28, 0.08)	0.50 (0.04, 0.96) *	−3.36 (−0.82, 0.10)
Anxiety and depression	−4.08 (−6.75, −1.40) **	−1.54 (−4.38, 1.30)	−1.30 (−2.80, 0.20)	−0.67 (−2.18, 0.85)
Adjusted R^2^	−	28.56%	−	16.28%
VIF	−	1.12−3.20	−	1.03−7.05
	**Colorectal Cancer (*n* = 451)**		**Prostate Cancer (*n* = 1105; Only Males)**	
	**Unadjusted Coefficient** **b (95% CI)**	**Adjusted Coefficient** **b (95% CI)**	**Unadjusted Coefficient** **b (95% CI)**	**Adjusted Coefficient** **b (95% CI)**
Male (reference: female)	−10.57 (−18.89, −2.25) *	−3.02 (−14.92, 8.89)	−	−
Age at accelerometer study	−2.16 (−2.76, −1.56) ***	−2.02 (−2.66, −1.38) ***	−2.31 (−2.76, −1.85) ***	−2.37 (−2.84, −1.90) ***
White (reference: non-white)	−10.49 (−40.13, 19.14)	−18.66 (−47.62, 10.30)	−9.91 (−28.81, 8.99)	−8.08 (−25.98, 9.82)
Townsend Index of Deprivation	−0.72 (−2.15, 0.72)	−0.52 (−1.87, 0.83)	0.27 (−0.77, 1.31)	0.18 (−0.81, 1.17)
BMI				
Underweight	−11.89 (−73.42, 49.63)	−25.84 (−85.79, 34.12)	48.89 (−39.50, 137.29)	62.0 (−21.24, 145.29)
Normal	0.00 (reference)	0.00 (reference)	0.00 (reference)	0.00 (reference)
Overweight	−11.19 (−20.67, −1.70) *	−5.85 (−15.07, 3.37)	−11.52 (−17.78, −5.27) ***	−7.95 (−13.97, −1.92) *
Obese	−25.21 (−36.41, −14.01) ***	−17.04 (−28.26, −5.82) **	−24.85 (−32.77, −16.94) ***	−17.97 (−25.92, −10.03) ***
Smoking status				
Never	0.00 (reference)	0.00 (reference)	0.00 (reference)	0.00 (reference)
Previous	3.88 (−4.70, 12.46)	10.80 (2.49, 19.12) *	−1.56 (−7.13, 4.00)	7.23 (1.87, 12.59) **
Current	−8.49 (−25.43, 8.44)	−3.02 (−18.96, 12.91)	−7.36 (−19.15, 4.43)	−5.90 (−17.06, 5.25)
Alcohol drinking frequency				
≤1–3 times/month	0.00 (reference)	0.00 (reference)	0.00 (reference)	0.00 (reference)
1–4 times/week	10.11 (−0.39, 20.61)	5.14 (−5.06, 15.34)	5.79 (−1.87, 13.45)	4.25 (−2.94, 11.45)
Daily or almost daily	7.25 (−4.07, 18.58)	6.07 (−5.19, 17.34)	4.55 (−3.76, 12.83)	2.44 (−5.45, 10.32)
Overall health rating				
Excellent	0.00 (reference)	0.00 (reference)	0.00 (reference)	0.00 (reference)
Good	−15.96 (−26.83, −5.08) **	−10.21 (−20.75, 0.32)	−8.23 (−14.89, −1.57) *	−4.22 (−10.72, 2.28)
Fair	−23.17 (−36.98, −9.37) **	−11.27 (−25.66, 3.12)	−23.22 (−31.88, −14.55) ***	−12.53 (−21.70, −3.35) **
Poor	−42.00 (−69.84, −14.17) **	−24.24 (−53.20, 4.71)	−49.46 (−73.73, −25.18) ***	−41.36 (−66.04, −16.67) **
No. of comorbidities	−9.69 (−15.12, −4.27) ***	−2.39 (−7.82, 3.05)	−11.34 (−15.00, −7.68) ***	−4.22 (−8.02, −0.42) *
Self-reported walking pace				
Slow	0.00 (reference)	0.00 (reference)	0.00 (reference)	0.00 (reference)
Steady or average	22.72 (7.14, 38.31) **	20.41 (3.93, 36.89) *	30.15 (15.16, 45.13) ***	14.57 (−0.09, 29.22)
Brisk	41.78 (26.00, 57.56) ***	31.01 (13.82, 48.20) ***	41.84 (26.85, 56.83) ***	21.34 (6.37, 36.31) **
Grip strength (kg)	−0.05 (−0.45, 0.34)	−0.27 (−0.85, 0.30)	0.18 (−0.15, 0.50)	−0.27 (−0.69, −0.05) *
Anxiety and depression	0.29 (−2.26, 2.84)	0.09 (−2.43, 2.60)	−0.28 (−1.93, 1.36)	0.56 (−1.05, 2.17)
Adjusted R^2^	−	18.44%	−	14.98%
VIF	−	1.09−5.15	−	1.01−9.03

Note: * *p* < 0.05, ** *p* < 0.01, *** *p* < 0.001. CI: confidence interval; BMI: body mass index.

**Table 4:** The relationship between pre-diagnosis MVPA (h/day) and all-cause death after cancer diagnosis (*n* = 2661).

	Unadjusted Hazard Ratio (95% CI) (*n* = 2661, No. of Events = 426)	Adjusted Hazard Ratio ^a^ (95% CI) (*n* = 2661, No. of Events = 426)
Time in MVPA (h/day)		
<1 h (reference)	1.0 (Reference)	1.0 (Reference)
1–1.5 h	0.63 (0.50, 0.81) ***	0.90 (0.69, 1.16)
1.5–2 h	0.49 (0.38, 0.64) ***	0.68 (0.52, 0.91) **
≥2 h	0.32 (0.24, 0.43) ***	0.57 (0.42, 0.78) ***
Cancer type		
Lung cancer (reference)		1.0 (Reference)
Female breast cancer		0.08 (0.05, 0.11) ***
Colorectal cancer		0.31 (0.24, 0.41) ***
Prostate cancer		0.08 (0.06, 0.10) ***
Male (reference: female)		1.25 (0.97, 1.62)
Age at cancer diagnosis		
<65 years		1.0 (Reference)
65–70 years		0.94 (0.68, 1.28)
70–75 years		1.25 (0.94, 1.66)
≥75 years		1.49 (1.08, 2.04) *
White race (reference: non-white)		1.58 (0.68, 3.65)
Townsend Index of deprivation		1.00 (0.96, 1.03)
BMI		
Underweight		3.24 (0.98, 10.66)
Normal		1.0 (Reference)
Overweight		1.04 (0.82, 1.32)
Obese		1.04 (0.79, 1.39)
Smoking status		
Never		1.0 (Reference)
Previous		1.18 (0.95, 1.47)
Current		1.47 (1.07, 2.02) *
Alcohol drinking frequency		
≤1–3 times/month		1.0 (Reference)
1–4 times/week		1.40 (1.08, 1.82) *
Daily or almost daily		1.25 (0.94, 1.67)
Diabetes		1.42 (0.97, 2.08)
Heart attack		0.77 (0.45, 1.32)
Angina		0.72 (0.42, 1.24)
Stroke		1.54 (0.87, 2.74)
Hypertension		0.94 (0.75, 1.17)
COPD		1.37 (0.85, 2.20)
Asthma		0.86 (0.63, 1.18)

Note. ^a^ adjusted for sex, age, race, Townsend Index of deprivation, BMI, smoking status, alcohol drinking frequency, and comorbidities. * *p* < 0.05, ** *p* < 0.01, *** *p* < 0.001. MVPA: moderate to vigorous physical activity; BMI: body mass index; CI: confidence interval; COPD: Chronic obstructive pulmonary disease.

## Data Availability

Data from the UK Biobank are available to researchers on application. This research has been conducted using the UK Biobank resource under application number 73792.

## Supplementary Materials

The following supporting information can be downloaded at: https://www.mdpi.com/article/10.3390/ijerph20021001/s1.

## Abbreviations

PA: physical activity; MVPA: moderate to vigorous physical activity; PACC: physical activity and cancer control; PHQ: Patient Health Questionnaire; SD: standard deviation; BMI: body mass index; CI: confidence interval.
