# Supplementary material for: Comparison of Pre-Diagnosis Physical Activity and Its Correlates between Lung and Other Cancer Patients: Accelerometer Data from the UK Biobank Prospective Cohort
Source: Int J Environ Res Public Health. 2023 Jan 5;20(2):1001. doi: 10.3390/ijerph20021001 (PMC9859178; doi:10.3390/ijerph20021001)
Supplement: Supplementary file 1 [file ijerph-20-01001-s001.zip › ijerph-2092985-supplementary.pdf]

Table 1. ICD-9 and ICD-10 codes.

Type of Cancer	ICD-9 and ICD-10 Codes
Lung cancer	C34.0 C34.1 C34.2 C34.3 C34.8 C34.9<br>1620 1622 1623 1624 1625 1628 1629
Breast cancer	C50.0 C50.1 C50.2 C50.3 C50.4 C50.5 C50.6 C50.8 C50.9<br>1740 1741 1742 1743 1744 1745 1746 1748 1749
Colorectal cancer	C18.0 C18.1 C18.2 C18.3 C18.4 C18.5 C18.6 C18.7 C18.8 C18.9 C19 C20<br>1530 1531 1532 1533 1534 1535 1536 1537 1538 1539 1540 1541
Prostate cancer	C61 185

Table 2. Correlates of time spent in MVPA (min/day) among lung, breast, colorectal and prostate cancer (Standardized coefficient).

	Lung Cancer	Breast Cancer	Colorectal Cancer	Prostate Cancer
	Beta	*Beta*	*Beta*	*Beta*
Male (reference: female)	0.02	–	−0.03	–
Age at accelerometer study	−0.34 ***	−0.26 ***	−0.30 ***	−0.29 ***
White (reference: non-white)	−0.01	−0.01	−0.06	−0.03
Townsend Index of Deprivation	−0.09	−0.04	−0.03	0.01
BMI				
Underweight	−0.07	0.04	−0.04	0.04
Normal	0.00 (reference)	0.00 (reference)	0.00 (reference)	0.00 (reference)
Overweight	−0.05	−0.10 **	−0.07	−0.09 **
Obese	−0.09	−0.13 ***	−0.16 **	−0.15 ***
Smoking status				
Never	0.00 (reference)	0.00 (reference)	0.00 (reference)	0.00 (reference)
Previous	−0.09	0.04	0.12 *	0.08 **
Current	−0.28 ***	−0.05	−0.02	−0.03
Alcohol drinking frequency				
≤1-3 times/month	0.00 (reference)	0.00 (reference)	0.00 (reference)	0.00 (reference)
1-4 times/week	0.02	0.09 *	0.06	0.05
Daily or almost daily	0.10	0.09 *	0.06	0.02
Overall health rating				
Excellent	0.00 (reference)	0.00 (reference)	0.00 (reference)	0.00 (reference)
Good	0.01	−0.01	−0.22	−0.05
Fair	−0.01	−0.05	−0.09	−0.10
Poor	−0.10	−0.07 *	−0.08	−0.10
No. of comorbidities	−0.07	−0.04	−0.04	−0.07 *
Self-reported walking pace				
Slow	0.00 (reference)	0.00 (reference)	0.00 (reference)	0.00 (reference)
Steady or average	0.14	0.19 *	0.23 *	0.16
Brisk	0.26 **	0.33 ***	0.34 ***	0.23 **
Grip strength (kg)	−0.15	−0.05	−0.06	−0.07 *
Anxiety and depression	−0.07	−0.03	0.00	0.02

Note. * $p < 0.05$, ** $p < 0.01$, *** $p < 0.001$.

Table 3. Interaction between cancer types and MVPA on all-cause mortality (n = 2,661).

	Adjusted Hazard Ratio [a] (n = 2,661, No. of events = 426)
Time in MVPA (hours/day)	
<1 hour (reference)	1.0 (Reference)
1-1.5 hours	0.93 (0.60, 1.43)

1.5-2 hours	0.82 (0.53, 1.27)
≥2 hours	0.72 (0.41, 0.24)
Cancer type	
Lung cancer (reference)	1.0 (Reference)
Female breast cancer	0.09 (0.05, 0.19) ***
Colorectal cancer	0.36 (0.23, 0.55) ***
Prostate cancer	0.09 (0.05, 0.15) ***
Cancer type # time in MVPA	
Female breast cancer × 1-1.5 hours	0.94 (0.39, 2.30)
Female breast cancer × 1.5-2 hours	0.86 (0.34, 2.17)
Female breast cancer × ≥2 hours	0.54 (0.20, 1.49)
Colorectal cancer × 1-1.5 hours	1.05 (0.57, 1.94)
Colorectal cancer × 1.5-2 hours	0.56 (0.28, 1.13)
Colorectal cancer × ≥2 hours	0.85 (0.41, 1.77)
Prostate cancer × 1-1.5 hours	0.78 (0.39, 1.58)
Prostate cancer × 1.5-2 hours	0.88 (0.43, 1.79)
Prostate cancer × ≥2 hours	0.69 (0.31, 1.53)

Note. [a] Adjusted for gender, age, race, Townsend Index of deprivation, BMI, smoking status, alcohol drinking frequency and comorbidities (diabetes, heart attack, angina, stroke, hypertension, COPD and asthma). *** $p < 0.001$.
